# A study on teachers’ classroom discourse strategies from the perspective of complex dynamic systems theory: lag sequential analysis based on pragmatic annotation and mental state transition

**DOI:** 10.3389/fpsyg.2026.1924971

**Published:** 2026-09-23

**Authors:** Jian Wang, Lina Wen

**Affiliations:** 1 School of Foreign Languages, Qingdao Agricultural University, Qingdao, Shandong, China; 2 School of Economics and Management (College of Cooperatives), Qingdao Agricultural University, Qingdao, Shandong, China

**Keywords:** adaptive timing awareness, complex dynamic systems theory, lag sequential analysis, sequence attractor, state space grid, teachers’ discourse strategies

## Abstract

Teachers’ classroom discourse is a key window into the quality of teaching. However, traditional research has mostly focused on the frequency distribution of discourse behaviors, overlooking the dynamic characteristics of discourse in the time dimension. From the perspective of Complex Dynamic Systems Theory (CDST), and using Lag Sequential Analysis (LSA) and State Space Grid (SSG) methods, this study examines four dynamic properties of the teacher discourse strategy system based on a video corpus of 72 lessons from 12 middle school teachers. First, historical dependence: teachers’ strategy selection is significantly constrained by the preceding three turns of the conversation. Second, adaptability: expert teachers identify shifts in students’ psychological states more sensitively and adjust strategies more quickly, with an adaptive switching latency of 2.1 s compared with 5.7 s for novice teachers. Third, nonlinearity: strategy diversity and student engagement show an inverted U-shaped relationship, with a “golden window” of entropy values between 1.32 and 2.78. Fourth, emergence: five recurring sequence patterns, interpreted here as candidate attractors, were identified; the “golden path of deep exploration” and “higher-order thinking path” characterize high-quality teaching, while the “deadlock pattern” signals declining teaching quality. The study offers an integrated methodological approach for operationalizing CDST in educational research and provides an empirical basis for teacher professional development and the design of classroom observation tools.

## 1 Introduction

As the main carrier of teaching and learning interaction, classroom discourse has long been regarded as a key window into the quality of teaching. Since Bellack et al. proposed language game analysis in the 1960s, classroom discourse research has undergone multiple paradigm shifts, from structuralist description to sociocultural interpretation (Weisi and Zandi, 2024). Researchers have accumulated a large amount of empirical evidence on teachers’ questioning, feedback, guidance and other behaviors (Sotelo et al., 2026). However, although existing research has been quite thorough in describing the distribution of discourse behavior types, it remains limited in explaining how these behaviors shape and constrain one another over time. When front-line teachers reflect on their own teaching, they often note that what makes a classroom feel “alive” or “dead” is not the use of isolated strategies, but whether the connections between strategies, and between teachers and students, are smooth and timely (Jutkowitz et al., 2026). This practical intuition points to something the traditional paradigm tends to overlook: classroom discourse is a complex system that evolves over time, whose meaning emerges from sequences and trajectories rather than from any single point of behavior. With the development of Complex Dynamic Systems Theory (CDST) in applied linguistics and education, researchers have begun to re-examine the temporal properties that traditional quantitative methods tend to filter out (Guo and Yu, 2025).

A small but growing body of work has begun to apply sequential and dynamic-systems thinking to classroom and educational interaction. Larsen-Freeman conceptualizes second language development as a non-linear, emergent process, while Hiver and Al-Hoorie introduce a dynamic systems perspective into the study of teachers’ cognition and motivation, documenting oscillation and attractor-like patterns over months and years of a teacher’s career (Yeh et al., 2026). These studies show that dynamic-systems concepts can be operationalized in language education research, but they operate mainly at a macro, biographical time scale and do not extend to the turn-by-turn structure of classroom talk itself. At the same time, sequence-oriented tools used elsewhere in classroom and collaborative-learning research have been able to identify local statistical dependencies between adjacent behaviors, yet they rarely integrate more than one dynamic property, historical dependence, adaptability, nonlinearity, and emergence, within a single analytic framework, and rarely connect these local dependencies to system-level, attractor-like structures. Teachers’ discourse strategy systems sit at this neglected intersection: they are directly observable at the turn level while carrying a rich temporal structure, making them a suitable setting in which to test the joint operationalization of these four properties (Li, 2026).

Although a variety of tools have emerged for measuring classroom interaction, including Sinclair and Coulthard’s IRF structure, Flanders’ interaction analysis system, Mercer’s inquiry-based conversation framework, and the T-SEDA tool developed by Hennessy et al., these tools have advanced both descriptive detail and coding precision, but they share an underlying assumption that classroom discourse can be treated as a set of independent behavioral units whose overall characteristics are captured by frequency or proportion (Hattan et al., 2026). This approach tends to erase dynamic properties such as sequential dependence, real-time adaptability, nonlinear transition, and system-level emergence, leaving classroom discourse research largely at a “snapshot” level and making it difficult to answer the more practical question of what kind of discourse sequence fosters deep learning (Soysal and Soysal, 2026). CDST offers a way to address this gap: it focuses on how a system’s state evolves over time, emphasizing four core properties, historical dependence (the current state is constrained by preceding states), adaptability (the system’s strategic response to external perturbation), nonlinearity (the relationship between variables need not remain proportional), and emergence (macro-level patterns arising from micro-level, self-organizing interaction) (Azlan and Goh, 2026). This study uses these four properties as analytical dimensions to reframe classroom discourse strategies from a “checklist” to a “system,” opening a more time-sensitive analytic space for discourse research, while combining this framework with lag sequential analysis (LSA) and state space grids (SSG) so that each property can be operationalized through a corresponding statistical or visual procedure rather than remaining a conceptual metaphor (Kibret et al., 2026).

Examining how these dynamic properties are expressed also raises the question of who exhibits them, and to what degree. Teaching expertise is not simply the accumulation of a larger inventory of strategies; prior CDST-informed research on teachers’ professional trajectories suggests that expertise is reflected in how flexibly and responsively a teacher’s discourse system reorganizes itself over time, from the more settled, attractor-like patterns documented in experienced teachers’ professional identities (Li, 2026) to the more exploratory, less settled patterns observed among novice teachers (Guo and Yu, 2025). Comparing expert and novice teachers therefore offers a natural, ecologically grounded test of the four dynamic properties: if historical dependence, adaptability, nonlinearity, and emergence are genuine features of a self-organizing discourse system rather than artifacts of the coding scheme, they should be expressed differently, and more distinctly, in teachers who differ in professional experience. This rationale motivates building the expert-novice contrast into each of the four research questions below, rather than treating it as a separate, additional analysis.

Building on this reasoning, this study uses lag sequential analysis (LSA) and state space grids (SSG) to examine four dynamic properties of the teacher discourse strategy system in middle school classrooms, and to explore how teaching expertise is reflected in these dynamic characteristics by comparing expert and novice teachers. The four research questions are organized around these properties, while remaining answerable through the specific statistical and visual procedures described in Section 3: RQ1, to what extent does a teacher’s current discourse strategy depend on the immediately preceding strategies, and is this dependence beyond what would be expected by chance? RQ2, how do teachers adjust their discourse strategies in response to changes in students’ psychological states, and do expert and novice teachers differ in this adaptive pattern? RQ3, is the relationship between strategy diversity and student engagement linear or nonlinear, and is there a threshold or optimal range? RQ4, what statistically significant sequential patterns characterize the joint teacher-student system, which of these recur stably enough to be treated as candidate attractors, and which are more transient?

## 2 Literature review and theoretical framework

### 2.1 Teacher classroom discourse strategies: classification systems and empirical findings

#### 2.1.1 Historical development of classroom discourse analysis

The academic pedigree of classroom discourse analysis can be traced to the analysis of “language games” proposed by Bellack et al. in 1966. This study pioneered the introduction of the concept of philosophy of language into the classroom, and constructed an analysis category of four types of teaching actions: structuring, soliciting, responding and reacting. Ten years later, Sinclair and Coulthard extracted the well-known IRF three-step structure in the Birmingham Discourse Model, namely teacher initiation, student response and teacher feedback, which profoundly shaped classroom discourse research over the following four decades with its concise and powerful generality (Riad, 2025). The Flanders interaction analysis system refines discourse behavior into ten categories and records it through time sampling, giving classroom behavior a basis for quantitative processing. In the 21st century, Alexander’s dialogic teaching framework, Mercer and Littleton’s distinction between exploratory and cumulative talk, Nystrand’s work on authenticity and “uptake,” and Hennessy’s recent T-SEDA observation tool have jointly pushed classroom discourse research from behavior counting toward dialogue quality (Detwiler, 2025). Across this line of tools, however, the analytic unit typically remains a discrete behavior or a short adjacency pair; even where categories capture the quality of an exchange, as in dialogic or exploratory-talk frameworks, they are still generally reported through frequency or proportion, and few studies track how a given sequence of behaviors persists, decays, or reorganizes over the course of a lesson. This leaves the temporal and sequential dimension of classroom discourse comparatively underexamined, which is the gap the present study is designed to address.

#### 2.1.2 Pragmatic dimensions of teacher discourse

By introducing speech act theory into classroom discourse research, researchers can attend simultaneously to the locutionary, illocutionary and perlocutionary dimensions of teacher talk (Moremi and Sekao, 2026). The classification of speech acts established by Austin and Searle provides an important reference for the functional analysis of classroom discourse, enabling researchers to distinguish teacher talk that shares a form but differs in function. The educational pragmatic labeling system recently proposed by Miura further operationalizes this dimension, incorporating strategies such as display questions, authentic questioning, elaboration, and metacognitive prompts into a unified coding framework, which provides a direct reference for the coding scheme design of this study (Rhemer et al., 2025).

### 2.2 Student psychological states and classroom interaction

#### 2.2.1 Classroom manifestation of cognitive states

The psychological states students experience in the classroom are far from captured by a simple dichotomy of “participation” versus “non-participation.” The concept of cognitive disequilibrium proposed by Piaget reveals the intrinsic tension learners experience when facing unexplained phenomena, which drives the formation of new cognitive structures (Aduko and Akayuure, 2025). D’Mello and Graesser’s continuum-based work on confusion, engagement and flow shows that moderate confusion can contribute to deep learning, while excessive confusion leads to frustration and disengagement (Nelen et al., 2025). Mercer’s distinction between exploratory talk and cumulative talk provides a detailed tool for analyzing the cognitive level of students’ discourse: the former involves reasoning, questioning and argumentation, while the latter centers on repetition, confirmation and accretion (Nguyen, 2025).

#### 2.2.2 Measurement of psychological state transitions

The measurement of mental states has long been constrained by methodological trade-offs. Traditional self-report scales are easy to implement but have low ecological validity and struggle to capture fleeting state changes in the classroom. Physiological indicators such as skin conductance and heart rate variability are more precise, but are highly invasive and difficult to deploy at scale in ordinary classrooms. Inference from behavioral indicators has gradually become a compromise solution, drawing on facial expressions, body posture, and discourse features (Mkimbili and Fredlund, 2025). Weighing feasibility against effectiveness, this study is positioned at the level of discourse cues, combining multi-dimensional features such as verbal hesitation, inferential markers, and confirmation signals to infer students’ psychological states (Tao et al., 2025).

### 2.3 Complex dynamic systems theory and its applications in educational research

#### 2.3.1 Core concepts of CDST

CDST constructs a distinctive epistemology around the core concepts of system, state, attractor, phase transition and historical dependence. A system is a whole composed of interacting components; a state is the configuration of the system at a given moment; an attractor is a state region the system tends to settle into or repeatedly re-enter; and a phase transition refers to the overall reorganization of the system under a change in external parameters (Dogbey, 2025). Scholars such as van Geert and Thelen have emphasized that time scale is a fundamental dimension of CDST analysis, since the dynamics observed at millisecond, minute, and yearly scales may present entirely different structures.

#### 2.3.2 Advances in CDST within applied linguistics and education

In applied linguistics, CDST was first introduced into second language acquisition research. Larsen-Freeman reconstructed language learning as a non-linear, self-organizing, and initial-condition-sensitive complex process. Hiver and Al-Hoorie extended this perspective to the study of teachers’ motivation and identity, revealing repeated oscillation and attractor structures in teachers’ professional lives. Stelma and Kostoulas proposed a CDST analysis framework for language teaching in 2024, calling for more researchers to move CDST from conceptual metaphor toward empirical operationalization (Yung, 2025). Much of this work, however, operates at the scale of a teacher’s motivation, identity, or career trajectory, measured in months or years; it has not yet been extended to the turn-by-turn scale of classroom discourse itself, where the same four properties, historical dependence, adaptability, nonlinearity, and emergence, arguably operate on a much shorter time scale but have not been systematically tested. This is the specific application gap that the present study is designed to fill, using classroom discourse as a setting in which CDST’s properties can be operationalized at the level of individual turns rather than career-long trajectories.

### 2.4 Lag sequential analysis and state space grids

#### 2.4.1 Methodological foundations of LSA

Lag sequential analysis was formally proposed by Sackett in 1978. Its core idea is to identify sequence transitions beyond the random level while controlling for the baseline probability of each behavior. The method first constructs a behavior transition frequency matrix, then calculates expected frequencies from the row and column margins, and finally judges the statistical significance of each transition through an adjusted residual. Its advantage over simple conditional probability is that it strips away the statistical illusion created by high-frequency behaviors and reveals the sequence structure that is genuinely binding. Educational researchers have applied LSA to classroom interaction, collaborative learning, and online discussion, accumulating relatively mature application experience (Yu and Huang, 2025).

#### 2.4.2 Theory and operation of SSG

The state space grid was developed by Lewis et al. Its basic idea is to track a system’s trajectory in a two-dimensional state space, characterizing the system’s stability and orderliness through indicators such as dwell time, visit frequency, and trajectory entropy (Moser and Zimmermann, 2025). This method is especially suitable for visualizing the joint evolution of two interacting variables, and can visually present the location of attractors and the moments of phase transition. In this study, LSA is used together with SSG: the former identifies statistically significant sequential patterns, and the latter visualizes and tracks these patterns within a two-dimensional state space (Yildiz and Osmanoglu, 2025).

### 2.5 Integrated theoretical framework: CDST discourse strategy model

The four research questions introduced in Section 1 are theoretically motivated by the four core CDST properties reviewed above: historical dependence and adaptability capture how the system behaves at the level of individual turns and short exchanges, whereas nonlinearity and emergence describe how these turn-level dynamics aggregate into broader patterns of engagement and recurring system states. Examining all four properties within a single study is necessary because they are conceptually interdependent, for example, a system with strong historical dependence but no adaptability would be rigid rather than genuinely dynamic, and because prior work, as reviewed above, has generally examined at most one of these properties in isolation.

Based on the above discussion, this study constructs a CDST discourse strategy integration model that takes the turn as the basic time unit, treats the teacher’s discourse strategy and the student’s psychological state as the two interacting dimensions of the system, and organizes historical dependence, adaptability, nonlinearity and emergence as the four analytical facets (Qiu, 2024). The model’s cross-scale logic is as follows: turn-taking at the micro level constitutes the basic event of the system, sequential patterns at the meso level constitute candidate attractors, and the relationship between diversity and participation at the macro level reflects the system’s overall nonlinear characteristics. The visualization of the theoretical framework is shown in Figure 1.

**Figure 1:**
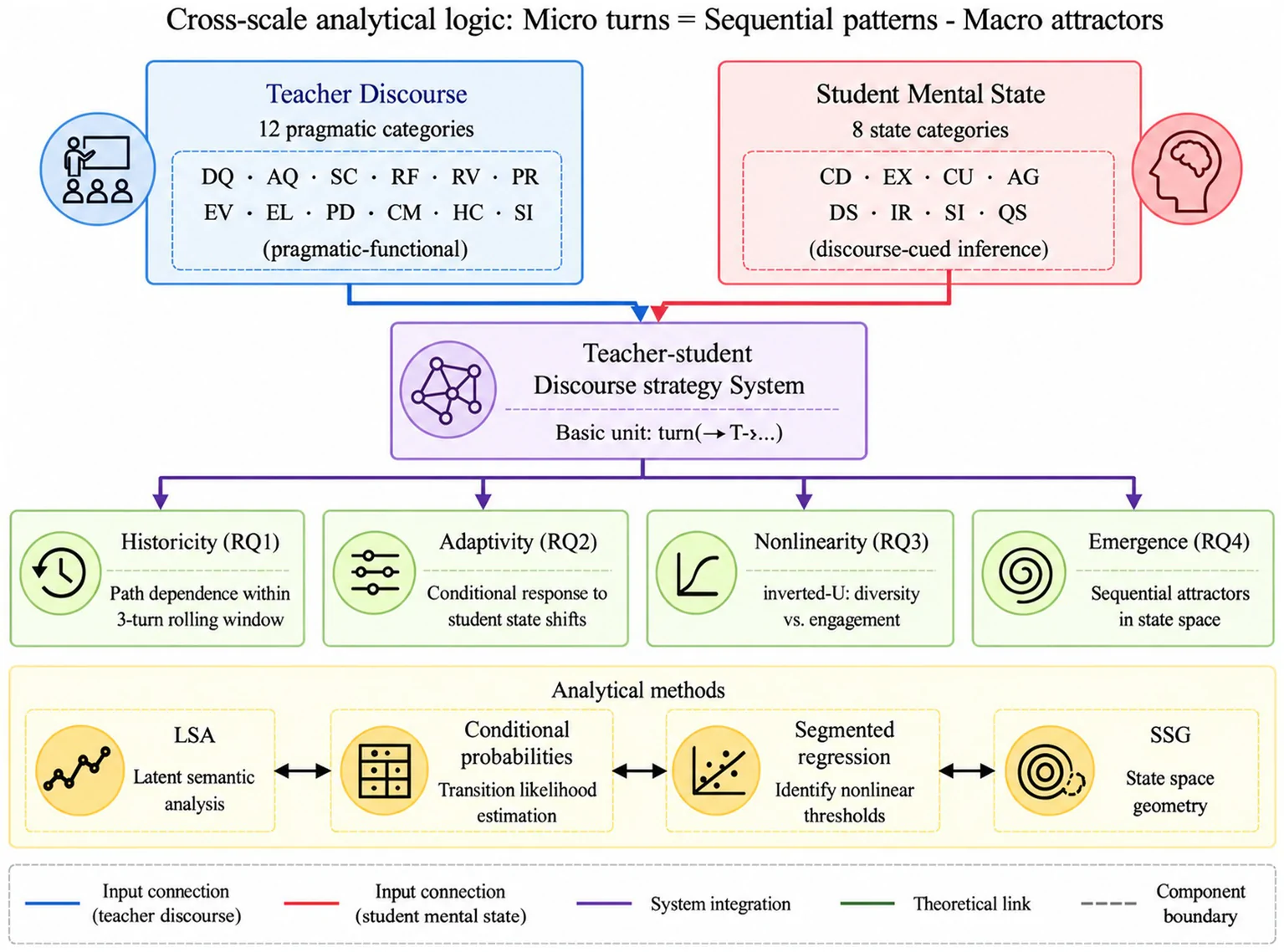
Conceptual model of teacher discourse strategy systems from a CDST perspective.

Each research question is aligned with one of the four dynamic attributes and draws on a consistent turn-based analytic unit, which supports comparability across the four analyses. It should be noted, however, that this alignment reflects analytic focus rather than a claim that each method fully establishes the corresponding theoretical property in the strict CDST sense; as discussed further in Sections 4 and 6, LSA and SSG provide statistical and visual evidence consistent with these properties, with CDST serving primarily as an interpretive lens for organizing and making sense of that evidence rather than as a framework the analyses directly validate.

## 3 Research methodology

### 3.1 Research design and methodological positioning

The study was designed as a sequential explanatory mixed-methods study, following the logical order of QUANT → qual, with quantitative analysis as the leading component and qualitative analysis as a supplement. The theoretical basis for this design is that the four dynamic attributes of CDST are highly quantifiable and need to be tested rigorously through statistical tools, while the interpretation of quantitative results needs to be enriched by returning to the classroom situation and teachers’ subjective experience. Specifically, LSA and SSG undertake the core task of identifying dynamic patterns, while thick description of classroom situations and retrospective interviews with teachers undertake the auxiliary task of explaining why these patterns occur. The overall research process is shown in Figure 2.

**Figure 2:**
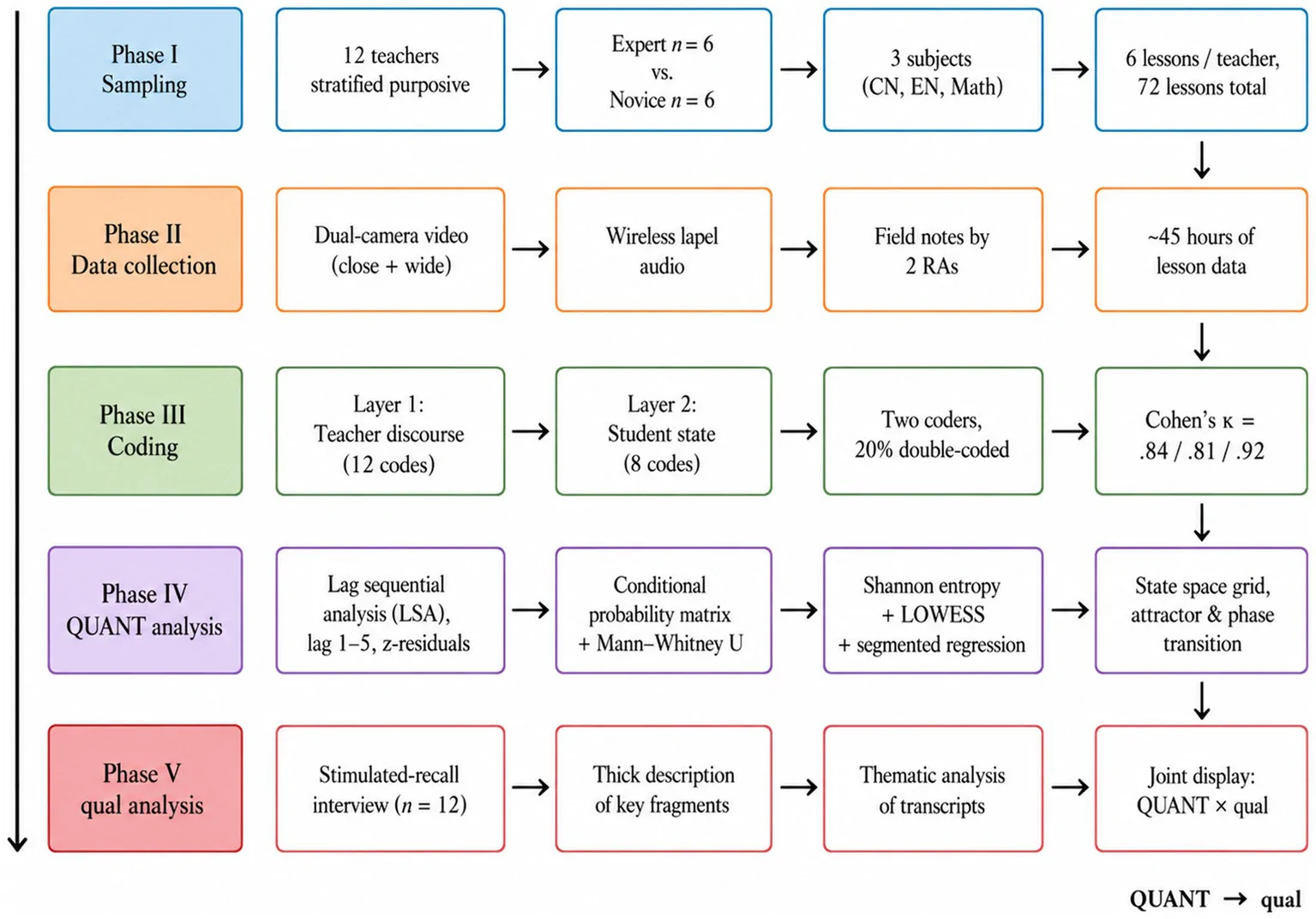
Research design and data flow process diagram.

### 3.2 Research participants and sampling

This study adopts a stratified purposive sampling strategy, selecting 12 teachers from two municipal demonstration middle schools in a provincial capital city as the research participants, including six expert teachers and six novice teachers. The teacher sample covers Chinese, English and mathematics, with four teachers per subject, two from the expert group and two from the novice group. The selection criteria for expert teachers were no less than 15 years of teaching experience and at least one municipal-level or higher teaching award. The selection criteria for novice teachers were no more than 5 years of teaching experience and no municipal-level or higher teaching award. Sampling within the same schools and subjects helps to control for potential interference from school culture and subject culture. The basic characteristics of the teacher sample are shown in Table 1.

**Table 1:** Basic information of research teacher sample.

ID	Subject	Group	Years of teaching	Gender	Highest degree	Teaching awards
T01	Chinese	Expert	18	F	Master’s	Provincial
T02	Chinese	Expert	22	M	Bachelor’s	Municipal
T03	Chinese	Novice	3	F	Master’s	None
T04	Chinese	Novice	2	F	Master’s	None
T05	English	Expert	16	F	Master’s	Municipal
T06	English	Expert	20	F	Master’s	Provincial
T07	English	Novice	4	M	Master’s	None
T08	English	Novice	1	F	Master’s	None
T09	Math	Expert	17	M	Master’s	Municipal
T10	Math	Expert	25	M	Bachelor’s	Provincial
T11	Math	Novice	5	M	Master’s	None
T12	Math	Novice	2	M	Doctorate	None

Each teacher’s six consecutive lesson videos were recorded to guard against anomalous fluctuations arising from incidental factors within a single lesson, resulting in a video corpus of 72 lessons totaling approximately 45 h. Lesson types included new instruction, review, and evaluation lessons, with proportions shown in Table 2.

**Table 2:** Distribution of classroom sample lesson types.

Lesson type	Number of lessons	Proportion (%)	Average duration (minutes)
New instruction	36	50.0	43.2
Review	24	33.3	41.8
Evaluation	12	16.7	42.5
Total	72	100.0	42.7

### 3.3 Data collection

Video recording used a two-camera scheme: one camera was placed at the back of the classroom to capture the teacher’s close-up, including facial expressions and verbal detail, and the other was placed at the front to capture students’ overall reactions and the classroom panorama. Teachers wore wireless lapel microphones to ensure their voices could be clearly captured even during group activities. In addition to video data, the study collected two types of supplementary data. The first is retrospective interviews: after preliminary data analysis, each teacher took part in a stimulated-recall interview of approximately 60 min, using key sequential fragments identified by LSA to prompt teachers’ reflection on their own discourse decision-making. The second is classroom observation notes: two research assistants recorded contextual information such as non-verbal interaction, seating adjustments, and use of teaching aids during each class, to support subsequent interpretation.

### 3.4 Coding scheme design

#### 3.4.1 Dual-layer coding architecture

The study constructed a dual-layer coding architecture: the first layer addresses teacher discourse strategies, differentiating 12 categories; the second layer addresses student psychological states, differentiating 8 categories. The teacher discourse strategy coding scheme appears in Table 3, and the student psychological state coding scheme appears in Table 4.

**Table 3:** Teacher discourse strategy pragmatic classification coding system.

Code	strategy category	Operational definition	Example utterance
DQ	Display Question	Teacher-anticipated answer question	Who is the author of “Silent Night Contemplation”?
AQ	Authentic Question	Open-ended teacher question without preset answer	How do you understand the emotional tone of this poem?
SC	Scaffolding Prompt	Providing clues to reduce task difficulty	Notice the opening of the third paragraph
RF	Restatement/Reformulation	Restating and semantically extending student response	So what you mean is…
RV	Echo/Repetition	Near-verbatim repetition of student’s own words (neutral)	Ah, feeling quite lonely
PR	Follow-up/Probe Question	Continuing questioning based on student response	Why do you think that way?
EV	Evaluative Feedback	Making a value judgment on the response	Good/Not quite correct
EL	Elaboration/Extension	Adding information based on student response	Yes, and furthermore…
PD	Directive/Procedural Discourse	Organizing classroom activities or behavior	Please turn to page 25
CM	Metacognitive Prompt	Guiding student reflection on thinking processes	How did you arrive at this conclusion?
HC	Humor/Affective Discourse	Non-content-related emotional regulation	This question has a bit of a trick to it
SI	Silence/Wait Time	Deliberate pause of ≥3 s	(silence)

**Table 4:** Student psychological state transition coding system.

Code	State category	Operational definition	Discourse/behavioral cues
CD	Cognitive disequilibrium	Manifesting confusion, uncertainty	Um/Furrowed brow/Hesitation
EX	Exploratory talk	Attempting reasoning, proposing hypotheses	Maybe it’s because…
CU	Cumulative talk	Repeating/confirming existing knowledge	Right, that’s what the textbook says
AG	Agreement/acceptance	Expressing agreement or understanding	Mm-hmm/Nodding
DS	Disagreement/questioning	Expressing objection or counterargument	But I think…
IR	Irrelevant/off-topic	Content unrelated to the lesson	Casual chat, distraction
SI	Silence/no response	Failure to respond within a reasonable time	Silence
QS	Student self-initiated question	Spontaneously posing a question	Teacher, but what if…?

The teacher discourse strategy coding scheme (Table 3) was adapted from Miura’s educational pragmatic labeling framework (Section 2.1.2), retaining categories such as display and authentic questions while adding categories specific to whole-class instruction that fall outside Miura’s original scope, namely directive/procedural discourse, humor/affective discourse, and silence/wait time, which are needed to describe classroom-management and affective functions of teacher talk that Miura’s original framework did not target. To avoid conceptual overlap between the two feedback-adjacent categories flagged during coder training, RF (Restatement/Reformulation) was operationally restricted to instances in which the teacher rephrases or semantically extends a student’s response beyond its literal wording (for example, adding precision or recasting register), whereas RV (Echo/Repetition) was restricted to near-verbatim repetition of the student’s own words without added propositional content. Coders were given explicit training examples distinguishing the two categories before formal coding began, and residual disagreements were resolved through the consultation procedure described in Section 3.4.2.

The eight categories in Table 4 combine indicators that sit at different conceptual levels: cognitive states (e.g., cognitive disequilibrium), discourse types (e.g., exploratory or cumulative talk), interactional stances (e.g., agreement or disagreement), and observable behaviors (e.g., silence). This scheme is therefore best understood as a discourse-cued proxy for students’ psychological engagement rather than a direct measure of their internal cognitive or affective states; codes were assigned from observable verbal and behavioral cues (Table 4, right-hand column), and results based on this coding should be interpreted as approximations of, rather than confirmed indicators of, students’ underlying psychological states. This limitation, and its implications for interpreting the adaptability and engagement findings, is discussed further in Section 6.

#### 3.4.2 Coding reliability

The study recruited two coders holding a master’s degree in education to carry out formal coding. Both coders received 40 h of coding training and then independently coded a random 20% sample to assess reliability. Cohen’s κ for teacher strategy coding reached 0.84, and κ for student state coding reached 0.81, both meeting the high-consistency standard recommended by Landis and Koch. For items with inconsistent coding, the study used a consultation mechanism in which the two coders discussed the item with the first author to reach consensus. Formal coding combined batch coding with regular reliability checks, with a spot-check review conducted every 15 classes to correct coding drift in a timely manner. Reliability indicators are shown in Table 5.

**Table 5:** Coding reliability statistical results.

Coding dimension	Consensus sample size	Cohen’s κ	95% confidence interval	Consistency level
Teacher discourse strategy	14 lessons	0.84	[0.81, 0.87]	High consistency
Student psychological state	14 lessons	0.81	[0.78, 0.84]	High consistency
Turn boundary determination	14 lessons	0.92	[0.90, 0.94]	Near-perfect agreement

### 3.5 Data analysis methods

Data analysis proceeded in four progressive stages: descriptive statistics, sequential analysis, targeted dynamic-property tests, and qualitative supplementation. The descriptive statistics stage calculated frequency distributions across strategy and state categories and constructed turn-based sequential data structures alternating between teacher and student behaviors. The sequential analysis stage built behavioral transition frequency matrices and computed adjusted residuals (z = (observed − expected) / √expected), using z > 1.96 (*p* < 0.05) and z > 2.58 (*p* < 0.01) as significance thresholds.

For the targeted dynamic-property tests: the analysis addressing historical dependence (RQ1) employed lag 1–5 autocorrelation analysis and chi-square comparison against randomly permuted sequences; the analysis addressing adaptability (RQ2) computed conditional probabilities P(teacher strategy j | student state i) and applied Mann–Whitney U tests to compare experts and novices on strategy repertoire breadth, switching latency, and related indicators; the analysis addressing nonlinearity (RQ3) operationalized strategy diversity via Shannon entropy and student engagement via cumulative proportions of exploratory talk, active questioning, and agreement states, using LOWESS smoothing curves and piecewise regression to identify breakpoints; and the state space grid analysis addressing emergence (RQ4) mapped 12-dimensional teacher strategies and 8-dimensional student states onto 96 potential cells, designated cells exceeding the mean dwell time by one standard deviation as candidate attractors, and computed trajectory entropy to reflect system order.

Qualitative supplementation followed the quantitative analyses, providing thick contextual description of key sequential episodes identified by LSA, conducting thematic analysis of teacher stimulated-recall interview transcripts, and integrating quantitative patterns with qualitative interpretations through joint displays.

## 4 Results

### 4.1 Descriptive statistics

#### 4.1.1 Overall distribution of discourse strategies

The 72-lesson corpus, after coding, yielded 26,847 instances of teacher discourse strategy and 21,392 instances of student psychological state. The teacher discourse strategy frequency distribution shows clear skewness: display questions, evaluative feedback, and directive discourse together account for 47.3% of all teacher discourse, while authentic questions, metacognitive prompts, and wait time together account for only 13.8%. Frequencies and proportions for all strategy types appear in Table 6.

**Table 6:** Teacher discourse strategy frequency and proportion.

Code	Frequency	Overall %	Expert group %	Novice group %
DQ	4,972	18.5	14.2	22.8
AQ	1,342	5.0	7.8	2.2
SC	2,389	8.9	11.3	6.5
RF	1,745	6.5	7.2	5.8
RV	1,611	6.0	5.4	6.6
PR	2,014	7.5	9.6	5.4
EV	4,109	15.3	12.1	18.5
EL	1,933	7.2	8.5	5.9
PD	3,766	14.0	11.8	16.2
CM	832	3.1	4.9	1.3
HC	1,235	4.6	4.7	4.5
SI	899	3.4	2.5	4.3
Total	26,847	100.0	100.0	100.0

The distribution of student psychological states likewise shows imbalance: agreement and cumulative talk together account for 51.4% of student discourse, while exploratory talk and self-initiated questions together account for only 18.7%. A preliminary comparison between expert and novice teacher classrooms shows significantly higher proportions of exploratory talk and cognitive disequilibrium states in expert teacher classrooms, with details in Table 7.

**Table 7:** Student psychological state frequency and between-group comparison.

Code	Total frequency	Overall %	Expert group %	Novice group %
CD	2,185	10.2	13.5	6.9
EX	2,934	13.7	18.2	9.3
CU	5,123	23.9	19.4	28.4
AG	5,876	27.5	23.7	31.2
DS	768	3.6	4.8	2.4
IR	985	4.6	3.1	6.1
SI	2,454	11.5	9.4	13.5
QS	1,067	5.0	7.9	2.2
Total	21,392	100.0	100.0	100.0

#### 4.1.2 Basic sequential characteristics

Each lesson averaged 668.4 turn-transition events. Average turn duration was 7.2 s in expert teacher classrooms and 9.8 s in novice teacher classrooms, a statistically significant difference (t = 4.32, *p* < 0.01). The ratio of teacher turns to student turns was 1.18:1 in the expert group and 1.86:1 in the novice group, indicating that discourse authority concentrates more heavily on the teacher in novice classrooms.

### 4.2 RQ1: sequential regularities in teacher-student strategy transitions

Consistent with the caution noted in Section 2.5, the patterns reported below reflect local, adjacency-based sequential dependence between nearby turns rather than a direct demonstration of path dependence across an entire lesson or a teacher’s longer developmental history. They are interpreted through the lens of CDST’s historical dependence property, and offer supporting evidence for that property at the level of adjacent-turn structure, without being read as establishing the property in its fuller theoretical sense.

#### 4.2.1 Lag-1 to Lag-5 transition probability matrices

Lag-1 LSA analysis identified 37 significant transition pairs with adjusted residuals exceeding 2.58, of which 22 transitions ran from teacher strategies to other teacher strategies and 15 ran from student states to teacher strategies. The most notable high-z significant transitions appear in Table 8.

**Table 8:** Primary significant transitions at lag-1 (z > 2.58).

Initiating behavior	Following behavior	Adjusted residual *z*	Frequency
DQ → Student CU	CU → DQ	7.82	412
AQ → Student EX	EX → PR	6.94	287
Student CD → SC	SC → Student AG	6.21	234
EV → DQ	DQ → Student CU	5.83	389
CM → Student EX	EX → EL	5.56	156
SI → Student SI	Student SI → RV	4.12	178

Each row summarizes a three-event chain (Initiating Behavior → intermediate step → Following Behavior). The reported adjusted residual (z) and frequency describe the second transition in the chain (the Following Behavior column), which was the statistically stronger and theoretically focal link in each chain; the initiating transition is shown for context but is not separately tabulated here.

#### 4.2.2 Evidence of path dependency

To test whether the sequence structure is truly non-random, the original sequence was randomly permuted 500 times, and the transition probability distribution of the permuted sequences was compared with that of the actual sequence. The chi-square test results showed a highly significant difference between the actual and random sequences at lags 1 to 3 (χ^2^(143) = 1827.4, *p* < 0.001), with the difference decaying rapidly to non-significance beyond lag 4. This result indicates that teachers’ current strategy choice is mainly constrained by the preceding three turns, with little detectable influence beyond four steps. This window provides direct empirical support for historical dependence at the local, adjacent-turn level, suggesting that teachers’ in-classroom decision-making is not made independently at each turn, but is embedded in a three-turn rolling memory window. A representative short cycle, DQ → CU → EV → DQ, recurred 2,384 times across the 72 lessons, forming a short recurring cycle that foreshadows the sequential patterns examined more formally in Section 4.5.

#### 4.2.3 Between-teacher differences

Comparing transition-graph out-degrees between teacher groups revealed an average out-degree of 5.7 for expert teachers’ strategy transition graphs, compared with 3.4 for novice teachers, a significant difference by independent-samples t-test (t = 3.81, *p* < 0.01). This difference indicates that expert teachers connect a greater diversity of subsequent strategies following each strategy type, with their strategy systems showing higher internal connectivity. Comparative transition structures between the two groups appear in Figure 3.

**Figure 3:**
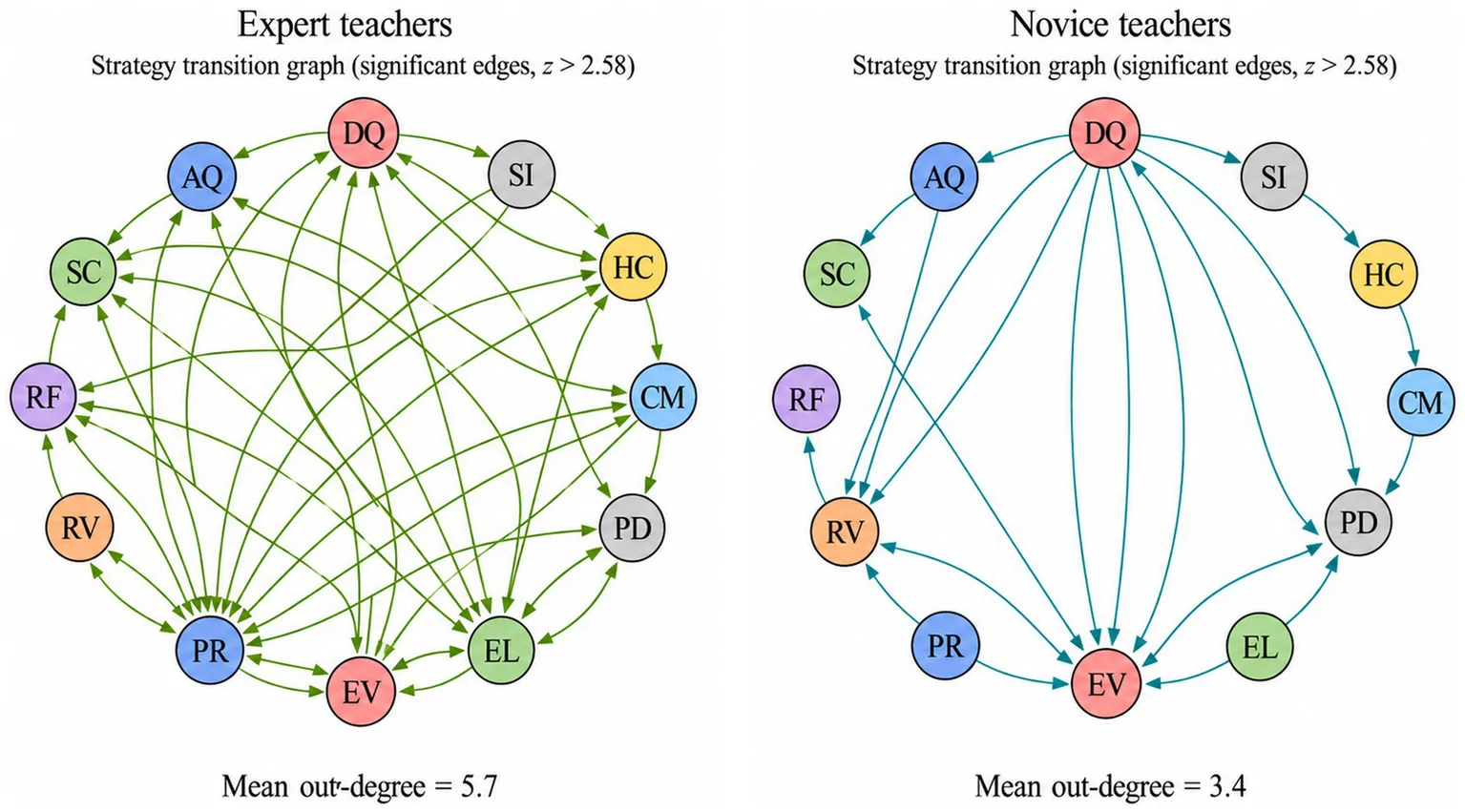
Comparative strategy transition diagrams of expert and novice teachers.

### 4.3 RQ2: teachers’ contingent responses to shifts in student state

#### 4.3.1 Conditional probability matrices

The study computed all conditional probabilities of P(teacher strategy | preceding student state) and visualized the resulting matrix as a heat map (Figure 4). Results show that expert teachers’ responses to students’ cognitive disequilibrium (CD) states concentrate on scaffolding prompts, with a corresponding probability of 0.41, while their responses to exploratory talk (EX) tend to sustain student reasoning through follow-up questions or metacognitive prompts, with a combined probability of 0.53. Novice teachers’ most frequent responses to cognitive disequilibrium involve providing evaluative feedback or directly repeating the question, with scaffolding prompts showing a probability of only 0.18.

**Figure 4:**
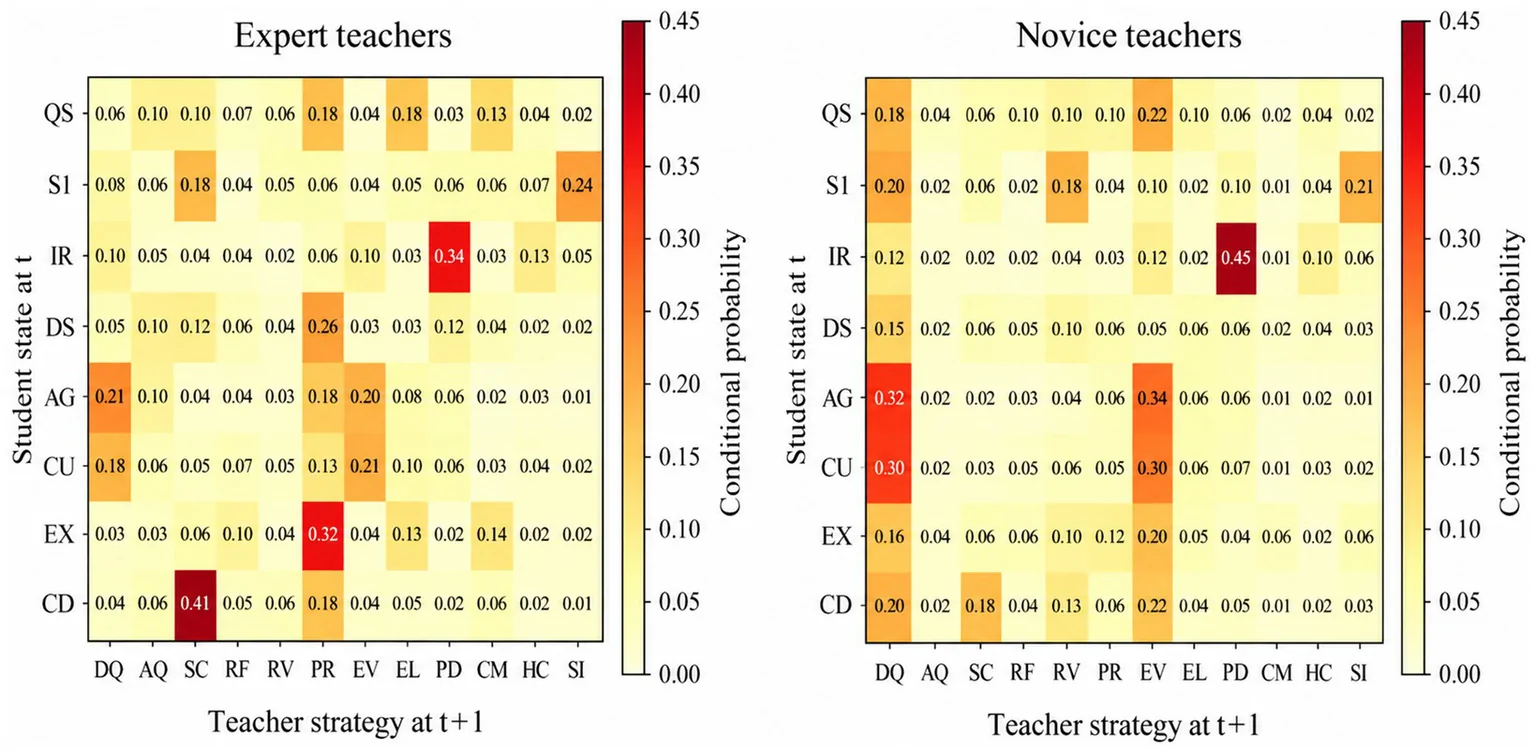
Heat map of conditional probability of teacher strategies in response to student states.

#### 4.3.2 Expert-novice adaptability comparison

A comparison of expert and novice teachers across four core adaptability indicators appears in Table 9. Mann–Whitney U tests showed that between-group differences on all four indicators reached statistical significance.

**Table 9:** Adaptability indicators comparison between expert and novice teachers.

Indicator	Expert group (*n* = 6)	Novice group (*n* = 6)	U value	*p* value	Effect size
Strategy repertoire breadth (number of categories)	9.8	6.3	2.5	<0.01	d = 1.42
Adaptive strategy switching latency (seconds)	2.1	5.7	1.0	<0.001	d = 1.83
Response probability to confusion signals	0.67	0.31	3.0	<0.01	φ = 0.36
Exploratory talk continuation rate	0.71	0.42	4.0	<0.05	φ = 0.29

Adaptive strategy switching latency, defined as the time interval between a student state change signal and the corresponding teacher strategy adjustment, represents one of the study’s key findings. Expert teachers averaged 2.1 s, compared with 5.7 s for novice teachers, a statistically significant difference. Expert and novice teachers also differed significantly in strategy repertoire breadth (9.8 vs. 6.3 categories, Table 9); however, the effect size for switching latency (*d* = 1.83) was considerably larger than that for repertoire breadth (*d* = 1.42). This pattern suggests that the expert-novice gap in adaptability is not fully explained by differences in the size of teachers’ strategy repertoire alone, but reflects, at least in part, teachers’ capacity to recognize a shift in student state and respond to it in a timely way.

### 4.4 RQ3: the relationship between strategy diversity and student engagement

#### 4.4.1 Distribution of strategy diversity

Shannon entropy indices across the 72 lessons ranged from 0.87 to 3.42, with a mean of 1.94 and a standard deviation of 0.61. Expert teacher classrooms averaged 2.31, while novice teacher classrooms averaged 1.52, a highly significant between-group difference (t = 5.23, *p* < 0.001). This result indicates that expert teachers markedly exceed novices in strategy-combination richness, though whether this advantage directly translates into higher student engagement required further testing.

#### 4.4.2 Inverted U-shaped curve

The pattern reported below describes the empirical, cross-sectional association between strategy diversity and student engagement across the 72 lessons; consistent with a nonlinear-systems perspective, it is interpreted as evidence suggestive of nonlinearity in this relationship, rather than as a direct, general-purpose test of nonlinearity as a systemic property.

The study plotted each lesson’s strategy diversity entropy against student engagement in scatter plots overlaid with LOWESS smoothing curves (Figure 5). Results show that the relationship between the two is not monotonically linear, but instead follows a pronounced inverted U-shape. Segmented regression identified two breakpoints, at H = 1.32 and H = 2.78, partitioning the diversity range into three segments: low-diversity, optimal, and overload zones, with regression parameters and mean student engagement for each segment shown in Table 10.

**Figure 5:**
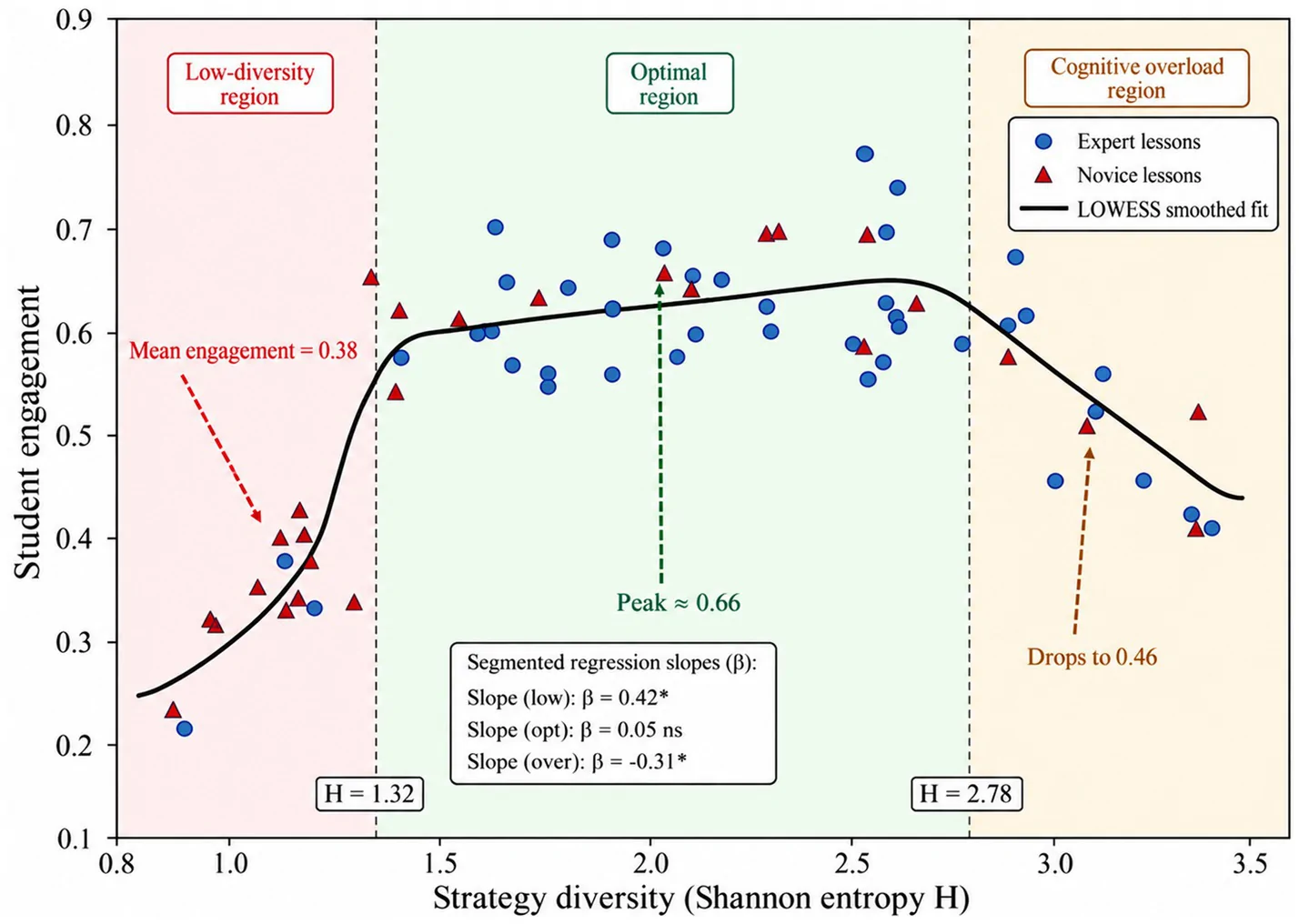
Scatter plot and LOWESS curve of strategy diversity and student engagement.

**Table 10:** Segmented regression results of strategy diversity.

Segment	Entropy range	Number of lessons	Mean engagement	Standard deviation	Regression slope
Low diversity zone	H < 1.32	18	0.38	0.07	*β* = 0.42*
Optimal zone	1.32 ≤ H ≤ 2.78	41	0.66	0.09	*β* = 0.05 (ns)
Overload zone	H > 2.78	13	0.46	0.11	*β* = −0.31*

Classrooms in the low-diversity zone are dominated by a singular “display question—evaluative feedback” cycle, with student engagement universally below 0.40. Classrooms in the optimal zone maintain a basic rhythm while introducing authentic questions, scaffolding prompts, and metacognitive prompts, achieving peak student engagement within this range. Classrooms in the overload zone feature rich strategy variety, but excessively frequent strategy switching appears to be associated with student cognitive overload, with engagement declining below 0.45.

### 4.5 RQ4: recurrent sequential patterns in the teacher-student system

#### 4.5.1 Strongest sequential patterns identified by LSA

Following convention in CDST-informed discourse research, the term “attractor” is used below to describe sequences that recur across the 72 lessons with a frequency and statistical strength exceeding chance expectation. This usage denotes a stable, recurring pattern in the observed data; it does not, on its own, establish the deeper dynamical-systems properties, such as basin stability or resistance to perturbation, that the term implies in its stricter mathematical sense.

LSA analysis identified five categories of recurring sequential patterns with adjusted residuals exceeding 3.5, detailed in Table 11.

**Table 11:** Primary recurring sequential patterns (candidate attractors) of the teacher-student system.

Rank	Significant sequence	*z* value	Frequency	Pattern interpretation
1	AQ → CD → SC → EX	6.84	147	Deep inquiry golden pathway
2	EV → DQ → CU → EV	5.21	234	Low-order consolidation cycle
3	CD → SC → AG → PR	4.93	98	Problem-solving pathway
4	CM → EX→EL → QS	4.57	76	Higher-order thinking pathway
5	SI → Student SI → RV → Student SI	3.82	112	Deadlock pattern (negative)

The top-ranked ‘AQ → CD → SC → EX’ sequence is termed the golden path of deep inquiry: teachers trigger students’ cognitive disequilibrium with authentic questions, help students cross this cognitive barrier through scaffolding prompts, and this in turn gives rise to exploratory talk. The frequency of this sequence was significantly higher in expert teachers’ classrooms than in novice teachers’ classrooms (108 vs. 39, *p* < 0.01). The second-ranked ‘EV → DQ → CU → EV’ sequence is termed the low-order consolidation cycle, characterized by teachers driving display questions with evaluative feedback, students responding with cumulative talk, and the cycle closing with further evaluation; this cycle is particularly common in novice teacher classrooms. The fifth-ranked ‘SI → Student SI → RV → Student SI’ sequence is termed the deadlock pattern, characterized by teacher silence triggering student silence; teachers attempt to break the impasse with an echo, but students remain silent again, forming a negative locking state within the system (Dinh and Nakatsubo, 2024).

#### 4.5.2 State space grid analysis

In this study, each lesson’s turn sequence was mapped onto a 96-unit state space grid, and candidate attractors were identified by cumulative dwell time. The SSG trajectory of the expert teacher’s classroom shows a more dispersed exploration pattern. The three cells with the highest dwell time are AQ-EX, SC-EX and PR-EX, together accounting for about 28% of the whole class (Figure 6).

**Figure 6:**
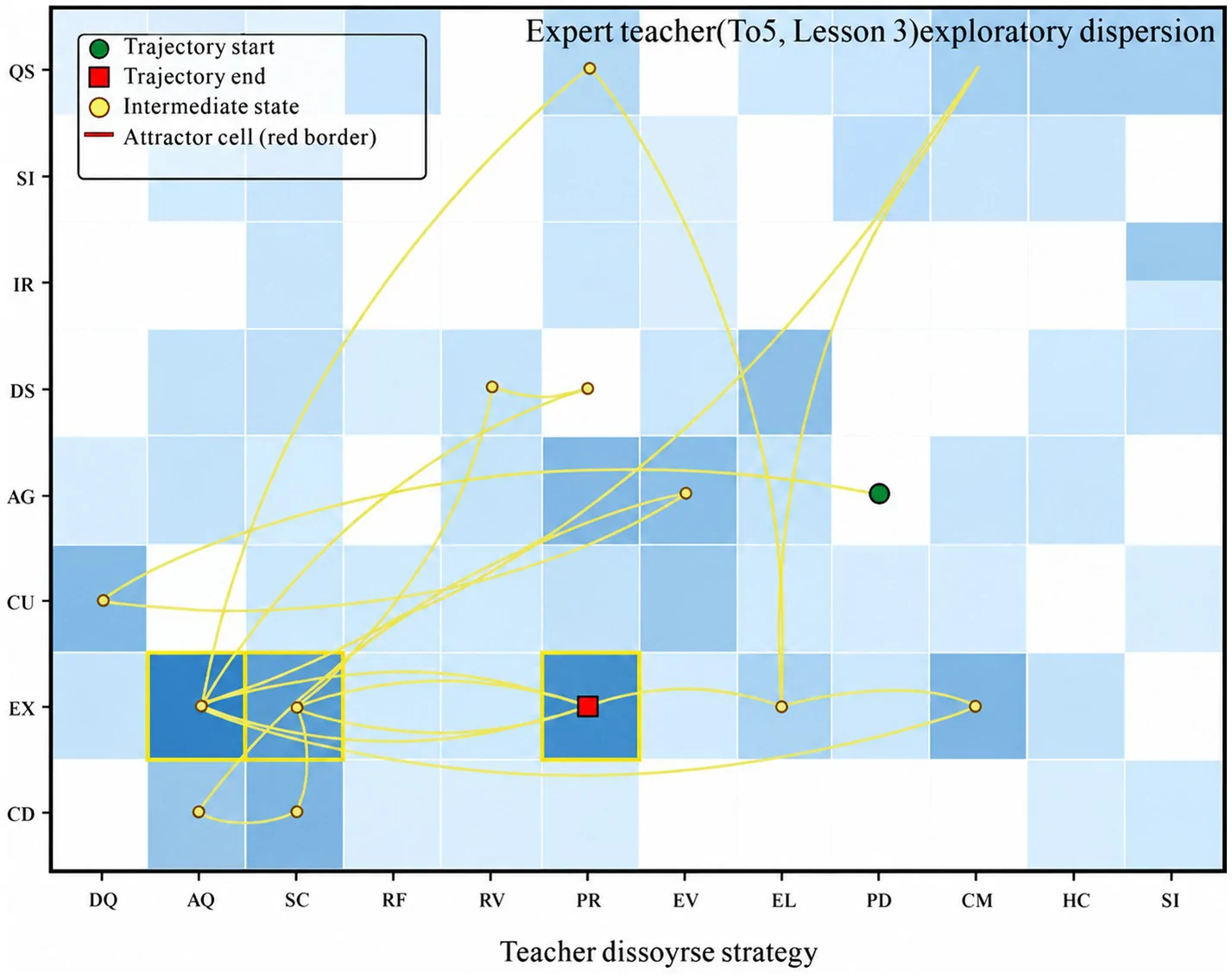
State space grid trajectory of a typical expert teacher classroom.

The SSG trajectory of the novice teacher’s classroom is more concentrated; the three cells with the highest dwell time are DQ-CU, EV-AG and PD-AG, together accounting for 41% of the total (Figure 7). This comparison intuitively illustrates a fundamental difference in behavioral patterns between the two teacher groups: the expert teacher’s system more often settles into the exploratory-attractor region, while the novice teacher’s system tends to remain locked in the IRF-cycle region.

**Figure 7:**
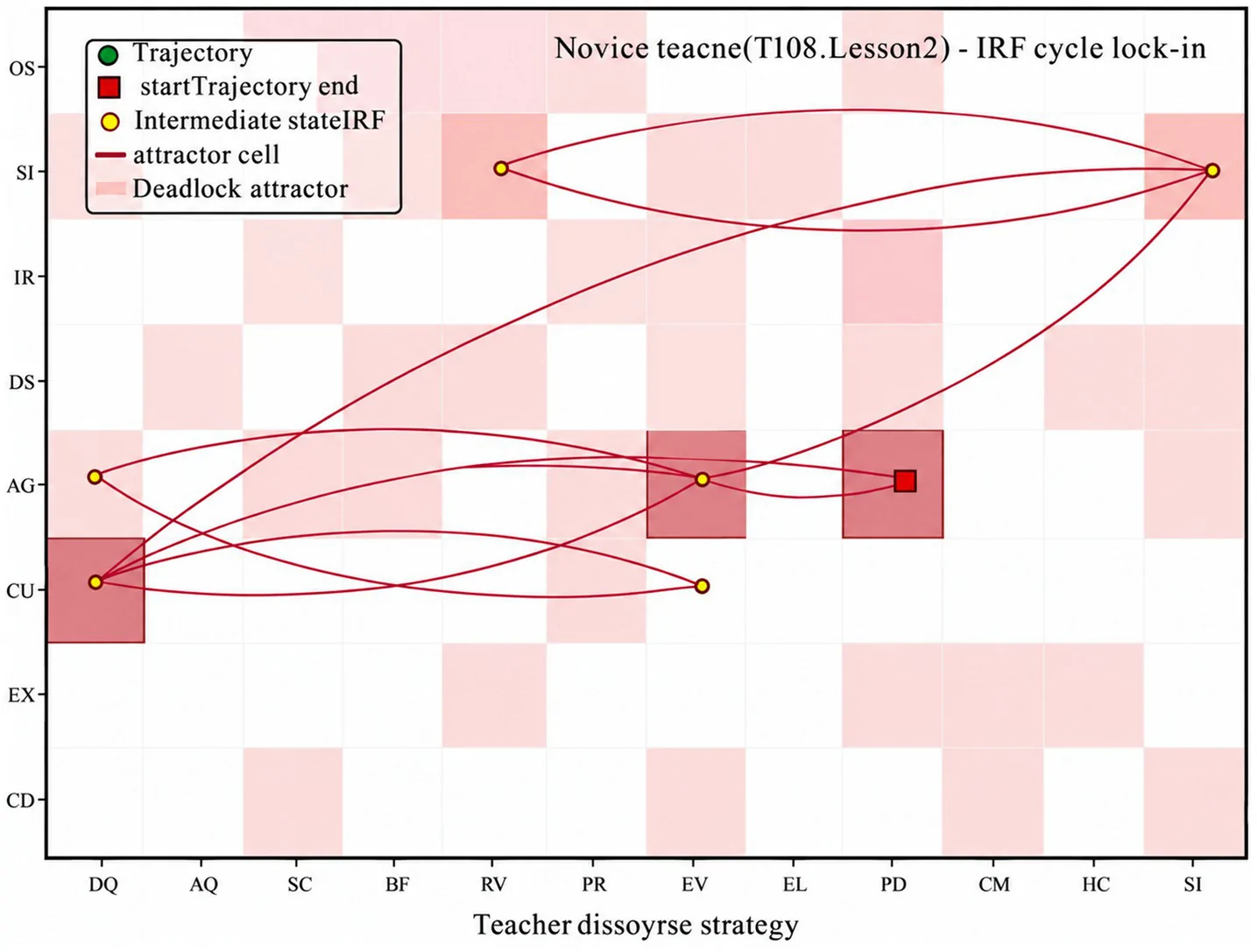
State space grid trajectory of a typical novice teacher classroom.

#### 4.5.3 Phase transition identification

Using rolling-window calculation of trajectory entropy for each lesson, the study identified state-jump phenomena at classroom teaching-stage transitions. Taking a Chinese-language new-lesson class as an example, the dominant attractor cell during the lesson-introduction phase was DQ-AG; upon entering the new-knowledge exploration phase, the system showed a marked transition around the twelfth turn, with the dominant attractor shifting to AQ-CD and SC-EX. Trajectory entropy before and after this transition jumped from 0.84 to 1.67. The identification of this phase transition provides direct, illustrative evidence consistent with CDST’s account of system reorganization. The overall phase-transition pattern is shown in Figure 8.

**Figure 8:**
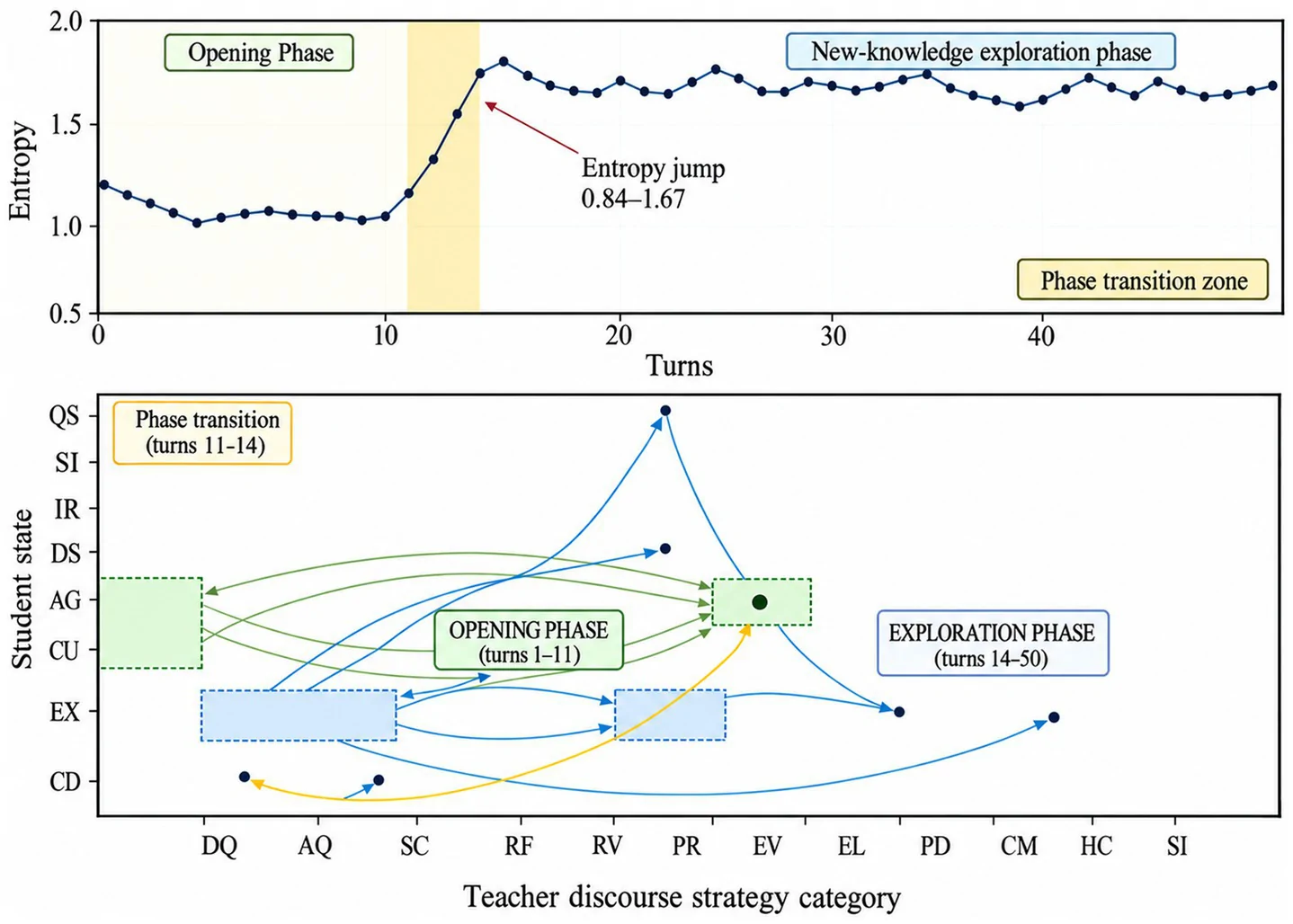
State space phase transition trajectory of a typical classroom.

### 4.6 Mixed methods integration: joint display

To integrate the key sequential patterns identified by LSA with explanatory data from teacher retrospective interviews, the study used a joint display strategy, positioning quantitative patterns, teachers’ own reflective interpretations, and representative segment identifiers within a single table (Table 12).

**Table 12:** Joint display of quantitative sequential patterns and teacher reflection.

Sequential pattern	Quantitative characteristics	Teacher reflective interpretation	Typical segment
AQ → CD → SC → EX	*z* = 6.84, high frequency in expert group	I observe students’ eye contact; when I detect confusion, I provide a hook	T05-L3-12 min
EV → DQ → CU → EV	*z* = 5.21, high frequency in novice group	Time is tight; I need to keep pace, so I work through foundational items first	T08-L2-25 min
CD → SC → AG → PR	*z* = 4.93, high frequency in expert group	Once resolved, I cannot stop; I must seize the moment to probe one level deeper	T01-L4-18 min
CM → EX → EL → QS	*z* = 4.57, high frequency in expert group	I guide him to reflect on his own thinking; naturally he’ll turn around and question me	T09-L5-30 min
SI → Student SI → RV → Student SI	*z* = 3.82, high frequency in novice group	At that moment I did not know what to do, so I just repeated it	T04-L1-22 min

The joint display shows that behind the statistical significance of the quantitative patterns lie teachers’ articulable practical wisdom, or practical dilemmas (Figure 9). Cross-validation between the quantitative and qualitative dimensions gives the study’s findings a more substantive explanatory basis (Figure 10).

**Figure 9:**
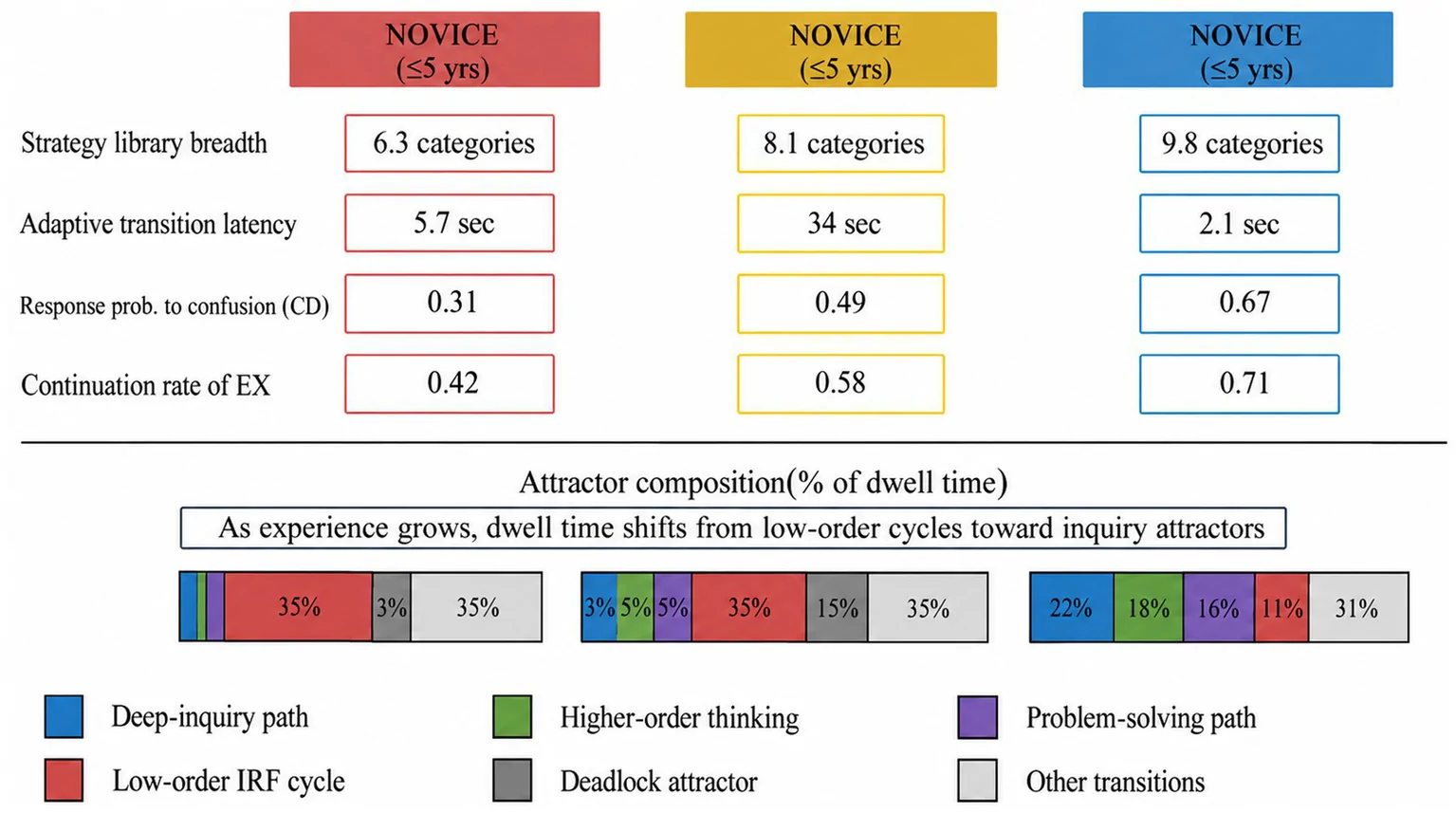
Schematic diagram of the association between teacher professional experience and attractor structures.

**Figure 10:**
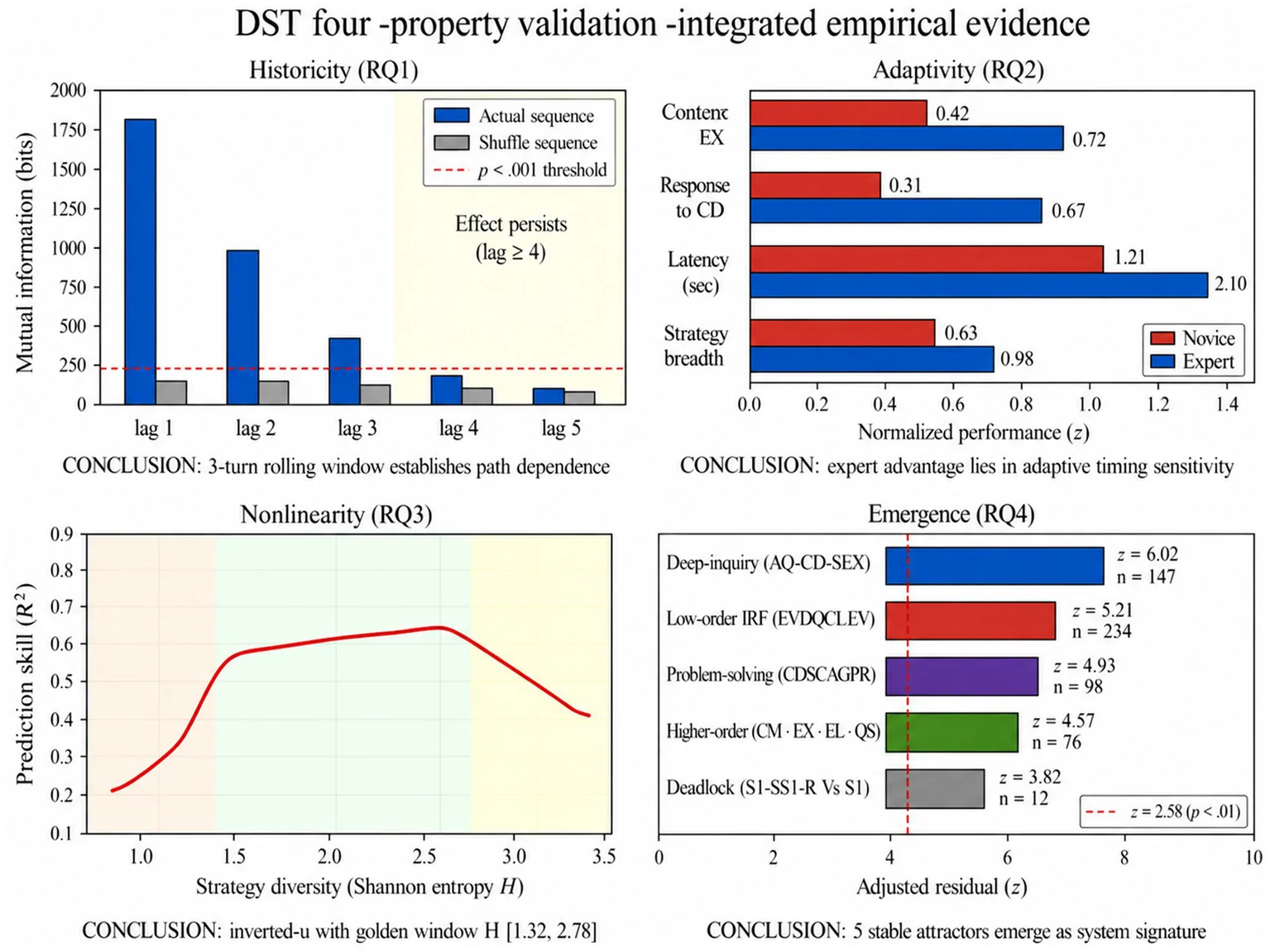
Integrated empirical evidence for the four CDST properties examined in this study.

The paragraphs below summarize the findings for each research question; the wording reports what the analyses show rather than treating each theoretical property as fully confirmed.

RQ1 (historical dependence): Lag 1–3 autocorrelation analyses and chi-square comparisons against randomly permuted sequences both showed highly significant differences between actual and random sequences (*χ*^2^(143) = 1827.4, *p* < 0.001), with the difference decaying to non-significance beyond lag 4. This pattern is consistent with a three-turn rolling window of local sequential dependence.
RQ2 (adaptability): Mann–Whitney U tests showed that expert teachers significantly outperformed novices on all four adaptability indicators: strategy repertoire breadth (9.8 vs. 6.3, *p* < 0.01), adaptive switching latency (2.1 s vs. 5.7 s, *p* < 0.001), response probability to confusion signals (0.67 vs. 0.31, *p* < 0.01), and exploratory talk continuation rate (0.71 vs. 0.42, *p* < 0.05), with switching latency showing the largest effect size.
RQ3 (nonlinearity): LOWESS smoothing curves and piecewise regression identified two breakpoints (H = 1.32 and H = 2.78), partitioning strategy diversity into a low-diversity zone (*β* = 0.42*), an optimal zone (mean engagement = 0.66), and an overload zone (*β* = −0.31*), consistent with an inverted U-shaped relationship and an entropy “golden window.”
RQ4 (emergence): LSA identified five recurring sequential patterns with adjusted residuals exceeding 3.5. The “deep inquiry golden pathway” (AQ → CD → SC → EX, z = 6.84) and the “higher-order thinking pathway” (CM → EX→EL → QS, z = 4.57) occurred at significantly higher frequencies in expert teacher classrooms, while the “deadlock pattern” (SI → Student SI → RV → Student SI, z = 3.82) was concentrated in novice classrooms, consistent with its interpretation as an early warning signal for declining interaction quality.

## 5 Discussion

### 5.1 Theoretical implications of core findings

The findings below are organized by dynamic property; for each one, we state the principal finding, relate it to prior research, offer a possible explanation, and note the study’s theoretical contribution. Taken together, the four sets of findings provide converging, though necessarily partial, empirical evidence consistent with CDST’s four dynamic properties, obtained, respectively, through LSA’s sequential analysis, SSG’s spatial tracking, and nonlinear curve testing.

The historical dependency findings show that teacher discourse strategies are not the product of independent, turn-by-turn decision-making, but are embedded within a three-turn rolling window. This resonates with van Geert’s notion of “soft assembly,” the idea that cognitive and behavioral systems perform moment-by-moment functional assembly based on current context and recent history rather than invoking fixed templates, and it instantiates this abstract concept as a measurable window width, repositioning teachers within a person-situation co-constructed cognitive system.

1. The adaptability findings suggest that expert-novice differences lie less in strategy-repertoire size than in timing sensitivity, in knowing “when to switch.” While echoing Schön’s classic account of the reflective practitioner, this study converts an implicit reflective capacity into an explicit temporal measurement through the adaptive switching latency indicator, and on this basis proposes “adaptive timing awareness” as a candidate new dimension of teacher professional knowledge (Li, 2023).
2. The nonlinearity findings, an inverted U-shaped relationship between strategy diversity and student engagement, challenge the intuitive assumption that more strategies are always better. Two complementary mechanisms may jointly account for the engagement decline observed in the overload zone: cognitively, excessive strategy switching may inflate students’ metacognitive monitoring load and displace content-processing resources; systemically, overly frequent changes may disrupt the stable interactional rhythm that a classroom, as a dynamic system, appears to require.
3. The emergence findings characterize the macro-structure of the classroom discourse system through five recurring sequential patterns. The deep inquiry golden pathway and the higher-order thinking pathway function as core recurring patterns of high-quality teaching; the over-representation of low-order consolidation cycles in novice classrooms signals a risk of entrenched instructional inertia; and the recurrent deadlock pattern functions as an early warning indicator of declining interaction quality, providing concrete diagnostic targets for classroom observation and teacher reflection.

### 5.2 Methodological contributions to CDST in educational research

CDST has long faced the dilemma of an “attractive theory but a difficult method” in education. Although researchers frequently invoke its concepts, they rarely integrate abstract properties such as historical dependence, adaptability, nonlinearity and emergence into a single empirical framework. This study offers a transferable operational scheme combining LSA, SSG and nonlinear curve testing. LSA quantifies the statistical significance of sequence dependence through adjusted residuals, turning the temporal property of historical dependence into a testable statistical hypothesis. SSG visualizes the system’s candidate attractor structure through trajectory tracking in two-dimensional state space, making the otherwise hard-to-describe concept of emergence more intuitively presentable. Nonlinearity testing refines the concept of nonlinearity into quantitative segmentation by identifying thresholds and a golden window through piecewise regression. This combination of methods is not limited to teacher talk research; it can be extended to a range of dynamic-system research settings, including teacher-student interaction, peer collaboration, and classroom management.

### 5.3 Dialogue with existing research

This study extends beyond the static description of the IRF structure found in traditional classroom discourse research. Sinclair and Coulthard’s IRF model reveals the pervasiveness of the classroom’s three-step structure, but does not explain how this structure evolves into deeper dialogue or degenerates into deadlock across different situations. This study’s attractor analysis responds to this open question: the deep inquiry pathway shows a specific mechanism by which IRF is extended into a four-step exploratory sequence, and the deadlock pattern shows a typical path by which IRF degrades into a repeating echo cycle. Nystrand’s research on authenticity and uptake emphasizes the importance of question type; this study further suggests that the function of a question type does not operate independently, but depends on its position and adaptive follow-through within the sequence. If an authentic question is not followed by supporting scaffolding and probing, its potential to trigger deep learning may not be realized (Jianbo, 2021).

In dialogue with teacher research from a CDST perspective, this study extends the research path Hiver and Al-Hoorie developed for teacher motivation to the level of teacher behavior. Research on teacher motivation focuses on oscillation and attractor structures at a macro time scale; this study brings that perspective down to the micro, second-by-second scale of turn-taking, addressing a gap in CDST-informed teacher research at this finer time scale. This study also responds to Stelma and Kostoulas's, 2024 call to move CDST research in language teaching from conceptual metaphor toward empirical operation, offering an empirical example in this direction.

### 5.4 Practical implications

These findings have direct implications for teachers’ professional development. Teacher training currently tends to treat the strategy list as its main content, asking teachers to master as many questioning skills, feedback types, and activity designs as possible. The nonlinearity findings here suggest that simply expanding this list may not necessarily improve teaching effectiveness. The focus of training might usefully shift from “strategy list” to “sequence awareness,” guiding teachers to attend to the connections between strategies, the coupling between strategies and students’ states, and the stability of strategy combinations over time. One concrete instrument is the personal transition map generated by LSA, which allows teachers to examine their own discourse decision-making habits visually, identify their dominant recurring patterns, and evaluate their instructional value. Training could also include practice in recognizing and breaking the “deadlock pattern,” so that teachers can intervene when an early signal of declining interaction quality appears.

These findings also have implications for classroom observation and evaluation. Traditional observation scales such as CLASS and FIAS take frequency and grade as their main measurement dimensions and largely omit temporal dynamics, making it difficult for them to capture the classroom’s character as a dynamic system. The “dynamic classroom observation protocol” envisioned in this study takes sequential patterns, phase-transition events, and adaptive latency as core indicators, offering a conceptual framework for designing the next generation of classroom observation tools.

These findings also carry reference value for the design of intelligent teaching systems. As deep-learning-based classroom speech transcription matures, it is technically feasible to embed an LSA-style algorithm into a real-time analysis module. When the system detects that a classroom has settled into an inefficient recurring pattern, it could unobtrusively surface adaptive scaffolding suggestions to teachers, achieving a low-interference technological enhancement of teaching.

## 6 Limitations and future research

This study has several limitations that should inform interpretation of its findings. First, the sample comprised 12 teachers from two demonstration schools in a single provincial capital, covering three subjects; while stratified sampling controlled for school and subject effects, the relatively small number of teachers per cell and the demonstration-school context limit how far the findings can be generalized to other regions, school types, or educational systems. Second, student psychological states were inferred from discourse and behavioral cues rather than measured directly (Section 3.4.1); this inference-based approach is feasible for large-scale classroom observation but cannot fully rule out misclassification of students’ actual internal states. Third, the analytic methods used here provide method-specific, partial evidence for each CDST property rather than a complete test of it: lag sequential analysis captures local, adjacency-based dependence rather than longer-range path dependence; segmented regression identifies a statistical association rather than a causal mechanism; and the sequential patterns labeled as attractors describe recurring empirical regularities rather than confirmed dynamical-systems attractors in the stricter mathematical sense. Future research could address these limitations through longitudinal designs that track the same teachers across a career-stage transition, larger and more geographically diverse samples, multimodal measures of student state that combine discourse cues with physiological or observational indicators, and experimental or quasi-experimental designs that manipulate strategy sequencing directly to test its causal effect on student engagement.

## 7 Conclusion

1. Drawing on approximately 45 h of classroom video from 12 secondary school teachers, and integrating lag sequential analysis, state space grid analysis, and nonlinear curve testing, this study examined four dynamic properties of the teacher discourse strategy system, interpreted through a CDST lens: historical dependence (strategy selection is significantly constrained by the preceding three turns); adaptability (expert teachers outperform novices on four indicators including strategy repertoire breadth and switching latency, with switching latency showing the largest effect); nonlinearity (an inverted U-shaped relationship between strategy diversity and student engagement, with an entropy “golden window” of 1.32–2.78); and emergence (five recurring sequential patterns, treated here as candidate attractors, with deep inquiry pathways marking high-quality interaction and deadlock patterns serving as an early warning signal).
2. Theoretically, this study jointly operationalizes all four CDST properties within a single analytic framework, addressing the tendency of prior research to examine at most one property in isolation; it proposes “adaptive timing awareness” as a candidate construct within teacher professional knowledge, translating a largely implicit reflective capacity into a measurable temporal indicator; and it offers an integrated LSA-SSG-nonlinear-testing approach as one operational pathway, among others future work might develop, for CDST-informed educational research. Practically, the study equips teacher professional development with sequence-analysis-based diagnostic tools, challenges the intuitive assumption that a larger strategy repertoire is always better, and positions the deadlock pattern as an early warning signal for declining interaction quality, together pointing classroom observation toward a greater awareness of sequence and timing.
3. Teacher discourse can be productively understood as a dynamic system evolving over time, rather than only as a checklist of strategies. How its history shapes the present, how it adapts to students’ states, when nonlinear transitions occur, and which patterns stabilize into recurring attractors are questions that matter both for CDST theory and for the practical improvement of teaching quality. This study offers an initial empirical map of these questions; the dynamic structures embedded in the classroom, one of the oldest and most complex sites of human collaboration, remain far from fully understood, and the deeper work of tracing them awaits future researchers.

## Data Availability

The original contributions presented in the study are included in the article/supplementary material, further inquiries can be directed to the corresponding author.
